# Supplementary material for: Population genetic structure of Patagonian toothfish (Dissostichus eleginoides) in the Southeast Pacific and Southwest Atlantic Ocean
Source: PeerJ. 2018 Jan 16;6:e4173. doi: 10.7717/peerj.4173 (PMC5774298; doi:10.7717/peerj.4173)
Supplement: Table S1 [file peerj-06-4173-s002.docx]

**Table S1.** Null allele frequency using Brookfield 1 formulae

|  | PN | PS | IQ | GP | PW | DR | IF | SGI |
| --- | --- | --- | --- | --- | --- | --- | --- | --- |
| **De4** | no | no | no | no | no | no | no | no |
| **De9** | no | no | 0.0698 | no | no | no | 0.0506 | no |
| **De30** | no | no | 0.0758 | no | no | 0.0692 | no | no |
| **De2** | no | no | no | 0.1492 | 0.0646 | no | no | no |
| **To2** | no | no | no | no | no | no | no | no |
| **To5** | no | no | no | no | no | no | 0.0361 | no |
