# Supplementary material for: Population genetic structure of Patagonian toothfish (Dissostichus eleginoides) in the Southeast Pacific and Southwest Atlantic Ocean
Source: PeerJ. 2018 Jan 16;6:e4173. doi: 10.7717/peerj.4173 (PMC5774298; doi:10.7717/peerj.4173)
Supplement: Table S2 [file peerj-06-4173-s003.docx]

**Table S2.** Details of parameters of genetic variability for each sample location and locus in *Dissostichus eleginoides*.

| Locality | Lat | Long | N | Parameter | De4 | De9 | De30 | De2 | To2 | To5 |
| --- | --- | --- | --- | --- | --- | --- | --- | --- | --- | --- |
| NP | 7°35’ | 81°15’ | 27 | N | 19 | 27 | 25 | 27 | 27 | 27 |
|  |  |  |  | N_A_ | 9 | 26 | 13 | 23 | 17 | 2 |
|  |  |  |  | H_O_ | 1 | 0.96 | 0.72 | 0.85 | 0.89 | 0.26 |
|  |  |  |  | H_E_ | 0.86 | 0.94 | 0.79 | 0.93 | 0.93 | 0.23 |
|  |  |  |  | HW | 0.67 | 0.93 | 0.01 | 0.11 | 0.73 | 0.44 |
| SP | 12°46’ | 77°27’ | 25 | N | 25 | 25 | 25 | 22 | 24 | 25 |
|  |  |  |  | N_A_ | 12 | 28 | 10 | 20 | 15 | 2 |
|  |  |  |  | H_O_ | 0.8 | 0.96 | 0.6 | 0.91 | 0.92 | 0.24 |
|  |  |  |  | H_E_ | 0.85 | 0.95 | 0.68 | 0.91 | 0.9 | 0.21 |
|  |  |  |  | HW | 0.01 | 0.49 | 0.05 | 0.16 | 0.91 | 0.5 |
| IQ | 20°16’ | 70°49’ | 42 | N | 42 | 41 | 31 | 41 | 42 | 42 |
|  |  |  |  | N_A_ | 11 | 27 | 13 | 19 | 18 | 3 |
|  |  |  |  | H_O_ | 0.91 | 0.81 | 0.55 | 0.93 | 0.93 | 0.12 |
|  |  |  |  | H_E_ | 0.88 | 0.94 | 0.68 | 0.91 | 0.92 | 0.11 |
|  |  |  |  | HW | 0.45 | 0 | 0 | 0.81 | 0.74 | 0.98 |
| GP | 48°52’ | 75°25’ | 24 | N | 24 | 24 | 16 | 14 | 24 | 24 |
|  |  |  |  | N_A_ | 12 | 21 | 8 | 10 | 16 | 2 |
|  |  |  |  | H_O_ | 0.96 | 0.96 | 0.56 | 0.57 | 0.96 | 0.04 |
|  |  |  |  | H_E_ | 0.87 | 0.94 | 0.55 | 0.85 | 0.91 | 0.04 |
|  |  |  |  | HW | 0.97 | 0.19 | 0.06 | 0.01 | 0.26 | 0.92 |
| PW | 54°92’ | 67°62’ | 54 | N | 54 | 48 | 53 | 37 | 54 | 54 |
|  |  |  |  | N_A_ | 12 | 24 | 11 | 14 | 21 | 2 |
|  |  |  |  | H_O_ | 0.85 | 0.9 | 0.64 | 0.68 | 0.96 | 0.06 |
|  |  |  |  | H_E_ | 0.87 | 0.92 | 0.66 | 0.79 | 0.92 | 0.05 |
|  |  |  |  | HW | 0.37 | 0.38 | 0.99 | 0 | 0.99 | 0.83 |
| DRI | 56°30’ | 68°37’ | 66 | N | 66 | 66 | 66 | 60 | 66 | 66 |
|  |  |  |  | N_A_ | 12 | 28 | 14 | 18 | 18 | 2 |
|  |  |  |  | H_O_ | 0.85 | 0.96 | 0.61 | 0.8 | 0.99 | 0.06 |
|  |  |  |  | H_E_ | 0.87 | 0.91 | 0.73 | 0.84 | 0.91 | 0.06 |
|  |  |  |  | HW | 0.07 | 0.64 | 0.11 | 0.3 | 0.24 | 0.8 |
| FI | 49°34’ | 54°28’ | 48 | N | 48 | 48 | 44 | 43 | 48 | 48 |
|  |  |  |  | N_A_ | 11 | 30 | 12 | 21 | 22 | 3 |
|  |  |  |  | H_O_ | 0.9 | 0.85 | 0.8 | 0.98 | 0.96 | 0.04 |
|  |  |  |  | H_E_ | 0.84 | 0.95 | 0.74 | 0.89 | 0.93 | 0.08 |
|  |  |  |  | HW | 0.99 | 0.81 | 0 | 0.41 | 0.12 | 0 |
| SGI | 54°39’ | 34°00’ | 71 | N | 60 | 68 | 61 | 71 | 71 | 71 |
|  |  |  |  | N_A_ | 11 | 17 | 11 | 17 | 18 | 2 |
|  |  |  |  | H_O_ | 0.97 | 0.99 | 0.74 | 0.92 | 0.85 | 0.1 |
|  |  |  |  | H_E_ | 0.76 | 0.7 | 0.65 | 0.82 | 0.87 | 0.09 |
|  |  |  |  | HW | 0.02 | 0.08 | 0 | 0.72 | 1 | 0.66 |
|  |  |  |  |  |  |  |  |  |  |  |
| CSA | NA | | 286 | N | 278 | 279 | 260 | 244 | 285 | 286 |
|  |  |  |  | N_A_ | 14 | 41 | 20 | 28 | 24 | 4 |
|  |  |  |  | H_O_ | 0.881 | 0.907 | 0.646 | 0.836 | 0.951 | 0.098 |
|  |  |  |  | H_E_ | 0.874 | 0.950 | 0.718 | 0.894 | 0.931 | 0.100 |
|  |  |  |  | HW | 0.047 | 0.000 | 0.000 | 0.000 | 0.792 | 0.000 |
| CGI | NA | | 71 | N | 60 | 68 | 61 | 71 | 71 | 71 |
|  |  |  |  | N_A_ | 11 | 17 | 11 | 17 | 18 | 2 |
|  |  |  |  | H_O_ | 0.967 | 0.985 | 0.738 | 0.915 | 0.845 | 0.099 |
|  |  |  |  | H_E_ | 0.761 | 0.703 | 0.653 | 0.824 | 0.865 | 0.094 |
|  |  |  |  | HW | 0.022 | 0.076 | 0.002 | 0.715 | 0.999 | 0.662 |
