# Supplementary material for: Population genetic structure of Patagonian toothfish (Dissostichus eleginoides) in the Southeast Pacific and Southwest Atlantic Ocean
Source: PeerJ. 2018 Jan 16;6:e4173. doi: 10.7717/peerj.4173 (PMC5774298; doi:10.7717/peerj.4173)
Supplement: Table S3 [file peerj-06-4173-s004.docx]

**Table S3.** Details of percentage of putative migrants and most likely origin area.

|  |  |  | Migration | | | | | | | | |
| --- | --- | --- | --- | --- | --- | --- | --- | --- | --- | --- | --- |
|  |  |  | MIG_PUT | Most likely area (%) | | | | | | | |
| Zone | Location | N | % | NP | SP | IQ | GP | PW | DRI | FI | SGI |
| Location | NP | 27 | 3.7 | **96.3** |  | 3.7 |  |  |  |  |  |
|  | SP | 25 | 8.0 |  | **92.0** |  | 4 |  | 4 |  |  |
|  | IQ | 42 | 4.8 | 2.4 |  | **95.2** |  | 2.4 |  |  |  |
|  | GP | 24 | 4.2 |  |  | 4.2 | **95.8** |  |  |  |  |
|  | PW | 54 | 1.9 |  |  |  | 1.9 | **98.1** |  |  |  |
|  | DRI | 66 | 3.0 |  |  |  | 1.5 |  | **97.0** |  | 1.5 |
|  | FI | 48 | 4.2 | 2.8 |  |  |  |  |  | **95.8** | 1.4 |
|  | SGI | 71 | 2.8 |  | 1.4 |  |  |  | 1.4 |  | **97.2** |
| Cluster | SAC | 286 | 0.7 | 99.3 | | | | | | | 0.7 |
|  | SGI | 71 | 11.3 | 11.3 | | | | | | | 89 |
