# Supplementary figures and images for: Population genetic structure of Patagonian toothfish (Dissostichus eleginoides) in the Southeast Pacific and Southwest Atlantic Ocean
Source: PeerJ. 2018 Jan 16;6:e4173. doi: 10.7717/peerj.4173 (PMC5774298; doi:10.7717/peerj.4173)

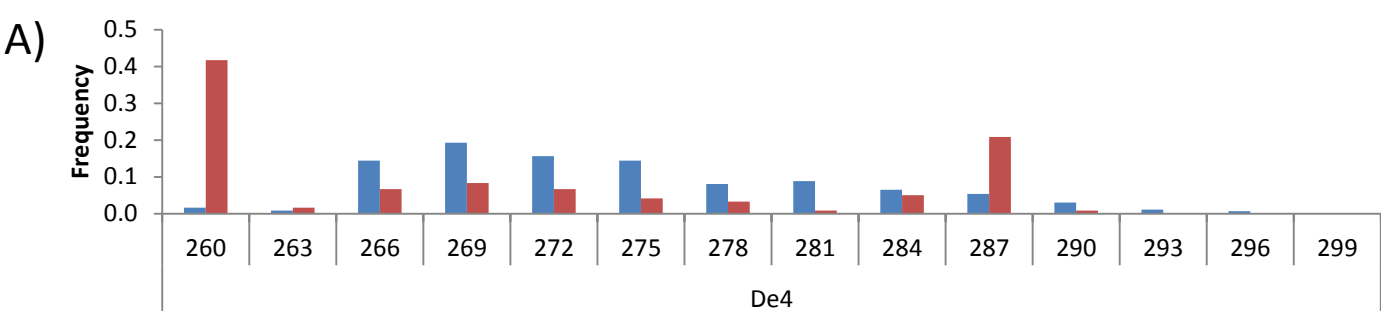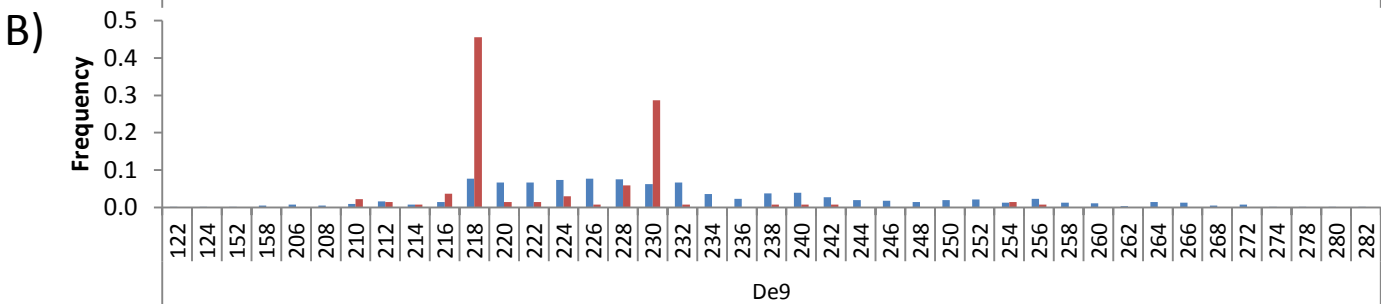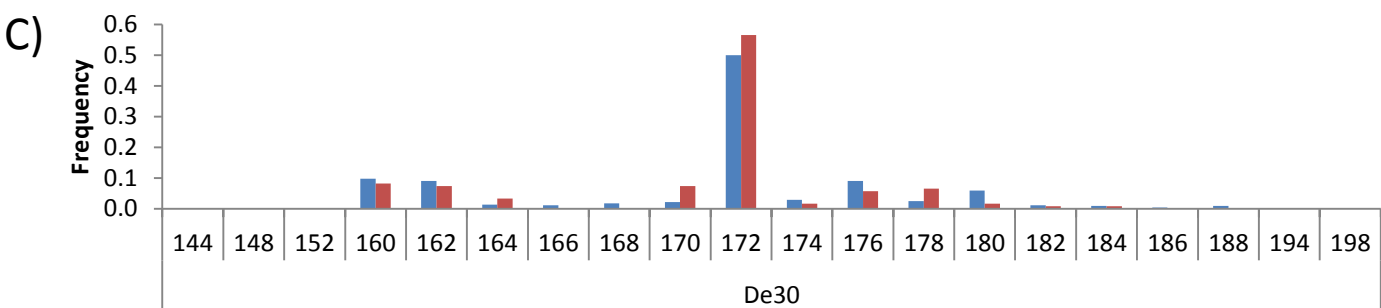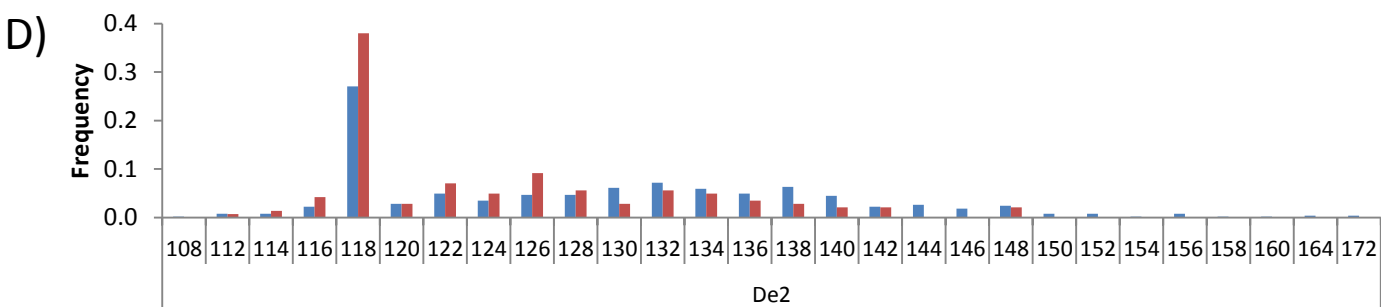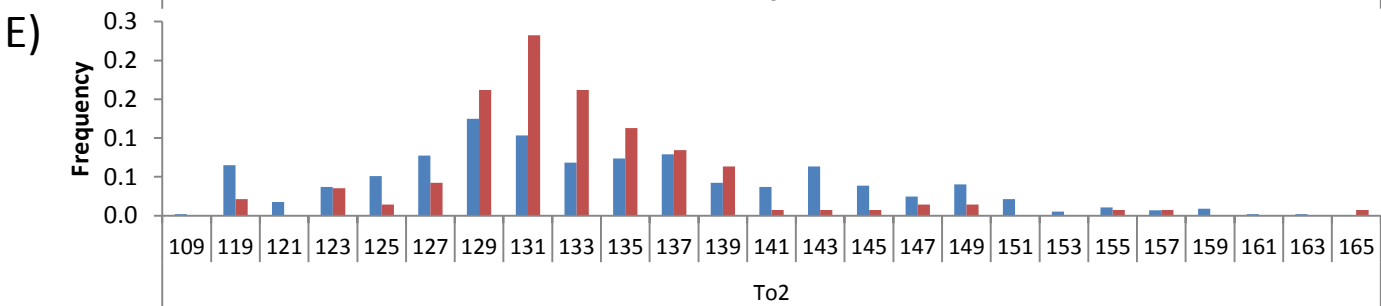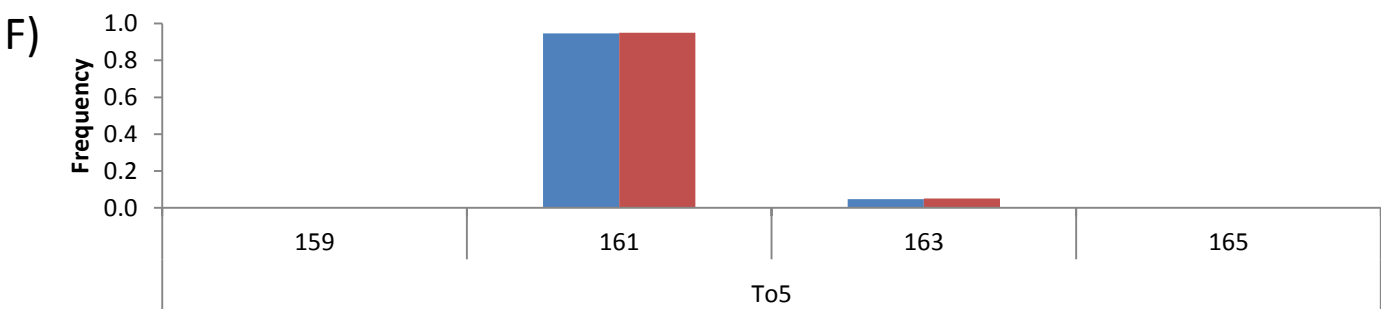

Supplement: Figure S3 — Blue bar correspond to South American Cluster (SAC) and red bar correspond to South of Georgia Cluster (SGC). [file peerj-06-4173-s007.pdf]
